# X-Inactive-Specific Transcript: Review of Its Functions in the Carcinogenesis

**DOI:** 10.3389/fcell.2021.690522

**Published:** 2021-06-11

**Authors:** Soudeh Ghafouri-Fard, Sepideh Dashti, Molood Farsi, Mohammad Taheri, Seyed Ali Mousavinejad

**Affiliations:** ^1^Department of Medical Genetics, Shahid Beheshti University of Medical Sciences, Tehran, Iran; ^2^Department of Genetics, Faculty of Biological Sciences, Tarbiat Modares University, Tehran, Iran; ^3^Urology and Nephrology Research Center, Shahid Beheshti University of Medical Sciences, Tehran, Iran; ^4^Skull Base Research Center, Loghman Hakim Hospital, Shahid Beheshti University of Medical Sciences, Tehran, Iran

**Keywords:** lncRNA, X-inactive-specific transcript, expression, biomarker, cancer

## Abstract

X-inactive–specific transcript (XIST) is one of the firstly discovered long non-coding RNAs with prominent roles in the process of X inactivation. Moreover, this transcript contributes in the carcinogenic process in different tissues. In addition to interacting with chromatin modifying molecules, XIST can be served as a molecular sponge for miRNAs to modulate expression of miRNA targets. Most of the studies have indicated an oncogenic role for XIST. However, in prostate cancer, a single study has indicated a tumor suppressor role for this lncRNA. Similar result has been reported for XIST in oral squamous cell carcinoma. In hepatocellular carcinoma, breast cancer, ovarian cancer, osteosarcoma, and renal cell carcinoma, different studies have reported inconsistent results. In the present manuscript, we review function of XIST in the carcinogenesis.

## Introduction

X-inactive–specific transcript (XIST) RNA is among the firstly discovered long non-coding RNAs (lncRNAs) in humans (Brown et al., 1992). The gene coding this lncRNA has at least eight exons and spans an area of about 17 kb on the X chromosome, in a region containing the X inactivation center (Brown et al., 1992). XIST RNA is primarily localized in the nucleus to a location not discriminable from the X inactivation-associated Barr body (Brown et al., 1992). The first important function attributed to XIST has been related to the process of X inactivation during which XIST induces gene silencing through recruitment of several chromatin modifying molecules (Loda and Heard, 2019). The indispensable role of Xist in X inactivation has been proved by targeted mutagenesis and transgenic experiments in mice showing skewing of this process following deletion of the *Xist* gene (Penny et al., 1996; Marahrens et al., 1997). Several molecules have been identified to interact with XIST to contribute in chromosome-wide gene silencing. SPEN, RBM15, WTAP, hnRNP K, and LBR are among molecules that participate in this process through interplay with XIST (Chu et al., 2015; McHugh et al., 2015). In addition, XIST has a prominent role in the carcinogenic processes. Several *in vitro*, *in vivo*, and clinical investigations have verified this aspect of XIST functions. In the present manuscript, we review function of XIST in the carcinogenesis.

## Cell Line Studies

### Breast Cancer

**FIGURE 1:**
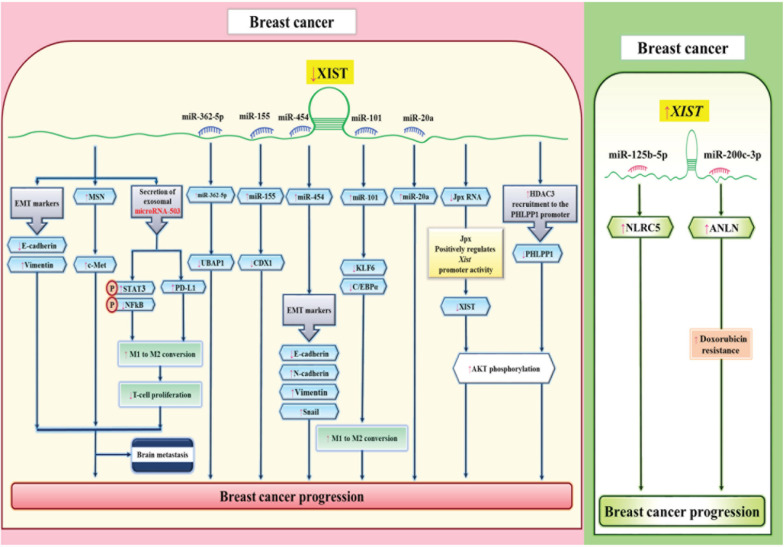
Different studies have shown the tumor suppressor role of XIST in breast cancer through sponging miRNAs (Li et al., 2020d; Liu et al., 2020) and regulating expression of epithelial-mesenchymal transition (EMT) markers (right panel). On the other hand, other studies have reported oncogenic roles for XIST (Zong et al., 2020) (left panel).

Functional impact of XIST in the breast carcinogenesis has been assessed in a number of *in vitro* studies. Liu et al. (2020) have reported down-regulation of XIST and UBAP1 in breast cancer cells. Forced up-regulation of XIST has attenuated proliferation, migration and invasion of these cells, and accelerated cell apoptosis. From a mechanistical point of view, XIST can interact with miR-362-5p and miR-362-5p to exert its effects. UBAP1 has been identified as miR-362-5p target, thus XIST modulates expression this protein via sponging miR-362-5p (Liu et al., 2020). Li et al. (2020d) have demonstrated down-regulation of XIST in triple negative breast cancer cells. Up-regulation of XIST has blocked cell proliferation and epithelial mesenchymal transition (EMT) while inducing apoptosis in these cell lines. miR-454 has been identified as a target of XIST in these cells (Li et al., 2020d). On the other hand, Zong et al. (2020) XIST has reported up-regulation of XIST in breast cancer cells, parallel with down-regulation of miR-125b-5p and up-regulation of NLRC5. XIST silencing has remarkably suppressed cell proliferation, migration, and invasion aptitudes of breast cancer cells. XIST has been shown to sponge miR-125b-5p and subsequently influence NLRC5 expression (Zong et al., 2020). Moreover, expression of XIST has been reported to be higher in doxorubicin-resistant breast cancer cells compared with parental cells. Furthermore, XIST up-regulation enhances cell proliferation and prohibited apoptosis of doxorubicin-treated breast cancer cells through enhancing expression of ANLN. XIST functions as a sponge for miR-200c-3p, which regulates expression of ANLN (Zhang et al., 2020). Figure 1 depicts different roles of XIST in the breast carcinogenesis.

### Gastric Cancer

**FIGURE 2:**
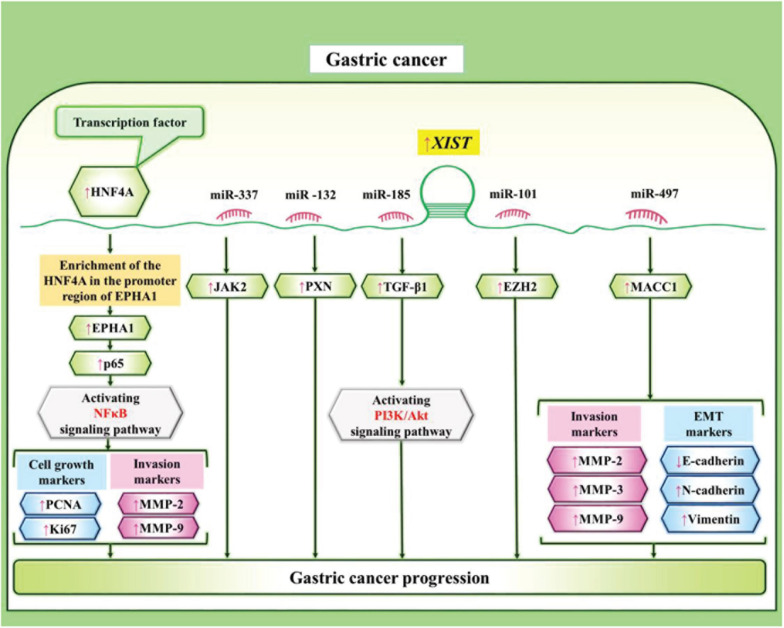
XIST can affect gastric carcinogenesis through sponging a number of miRNAs, thus regulating NF-κB and PI3K/AKT pathways (Chen et al., 2016; Lu et al., 2017; Ma et al., 2017; Zhang et al., 2018; Li et al., 2020a; Zheng et al., 2020).

In gastric cancer, XIST has been shown to exert oncogenic effects. Zheng et al. (2020) have demonstrated over-expression of XIST and down-regulation of miR-337 in these cells. XIST silencing has simultaneously suppressed proliferation, invasion, and migration of gastric cancer cells. Mechanistically, XIST increases expression of JAK2 through sponging miR-337 (Zheng et al., 2020). Consistently, over-expression of XIST in gastric cancer cells has been accompanied by up-regulation of PXN while down-regulation of miR-132. Furthermore, both XIST silencing and miR-132 over-expression could inhibit gastric cancer cell proliferation and migration (Li et al., 2020a). In this kind of cancer, XIST has also been shown to promote cell cycle progression at G1/S phase and block cell apoptosis through repressing miR-497 expression and up-regulating MACC1 levels (Ma et al., 2017). XIST can also sponge miR-185 to influence expression of TGF-β1 in gastric cancer cells (Zhang et al., 2018). Figure 2 depicts the role of XIST in gastric carcinogenesis.

### Colorectal Cancer

**FIGURE 3:**
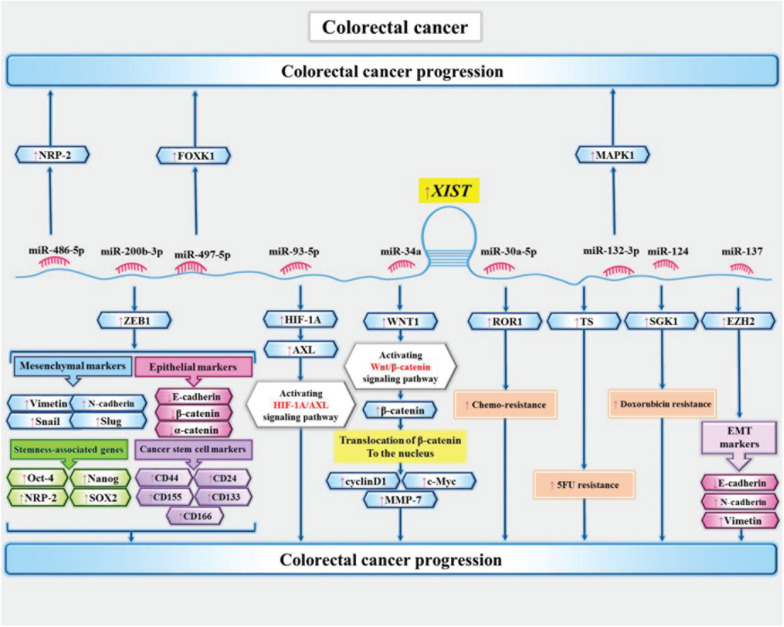
Downstream targets of XIST in colon cancer cells (Chen et al., 2017; Song et al., 2017; Xiao et al., 2017; Liu et al., 2020; Sun et al., 2018; Liu et al., 2019; Wang et al., 2020b). XIST enhances proliferation and epithelial–mesenchymal transition of colon cancer cells via sequestering miR-486-5p and increasing expression neuropilin-2 (Liu et al., 2019). It can also sequester miR-137 and subsequently increase expression of EZH2 to enhance metastatic ability of colon cancer cells (Liu et al., 2018).

Expression of XIST has been increased in colon cancer cells. Mechanistically, XIST sponges miR-34a and increases expression of WNT1. XIST also affects expression of β-catenin, cyclinD1, c-Myc, and MMP-7 in colon cancer cells (Sun et al., 2018). Moreover, expression of this lncRNA has been up-regulated 5-Flurouracil-resitant colon cancer cells. XIST silencing has inverted resistance phenotype in these cells. XIST has been shown to promote expression of thymidylatesynthase, an enzyme which is targeted by 5-Flurouracil (Xiao et al., 2017). Another study in colon cancer cells has demonstrated over-expression of XIST and FOXK1, while down-regulation of miR-497-5p. This study has also confirmed the significance of XIST/miR-497-5p/FOXK1 in the pathogenesis of colon cancer (Wang et al., 2020b). Figure 3 depicts the downstream targets of XIST in colon cancer cells.

### Pancreatic Cancer

**FIGURE 4:**
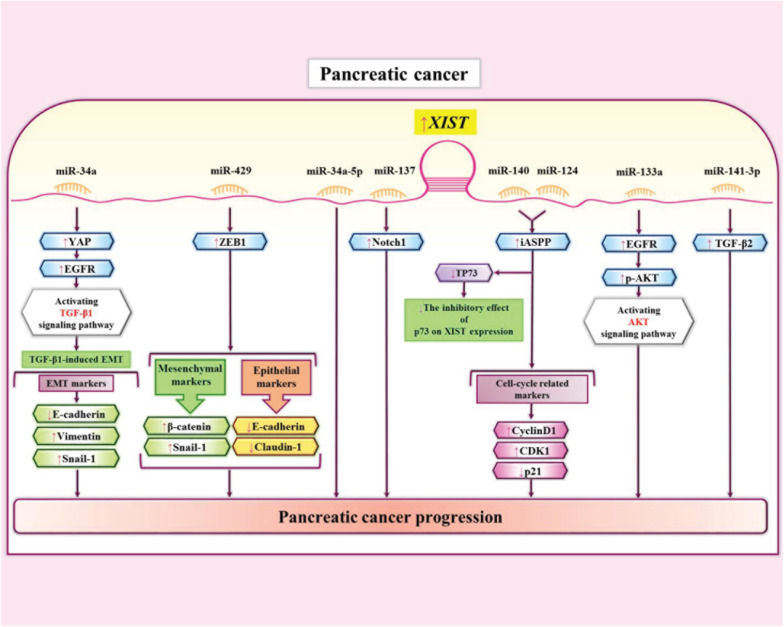
The interaction between XIST and miRNAs as well as their targets in pancreatic cancer cells (Liang et al., 2017; Wei et al., 2017; Sun et al., 2018; Shen et al., 2019; Sun and Zhang, 2019; Liu et al., 2020; Zou et al., 2020). XIST interaction with miR-140 and miR-124 increases expression of iASPP and promotes growth of pancreatic cancer cells (Liang et al., 2017). XIST also enhances expression of TGF-β2 through sequestering miR-141-3p. This interaction enhances invasiveness of pancreatic cancer cells (Sun and Zhang, 2019).

XIST has also been up-regulated in prostate cancer cell lines where it enhances their proliferation, migration and invasion, and suppresses cell their apoptosis. These effects are exerted through sponging miR-34a-5p (Sun et al., 2018). In these cells, XIST has also interactions with miR-137 through which it regulates expression of Notch1 (Liu et al., 2020). miR-141-3p is another miRNA which has been shown to be sponged by XIST in pancreatic cancer cells. XIST enhances expression of TGF-β2 through interacting with this miRNA (Sun and Zhang, 2019). Figure 4 depicts the interaction between XIST and miRNAs as well as their targets in pancreatic cancer cells.

### Bladder Cancer

**FIGURE 5:**
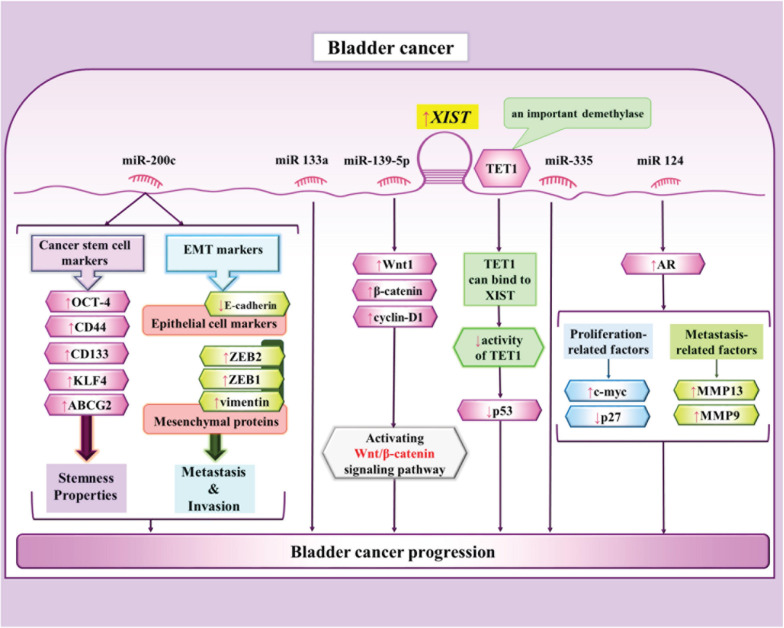
The interactions between XIST and miRNAs in bladder cancercells (Hu et al., 2017, 2019; Xiong et al., 2017; Xu et al., 2018; Zhou et al., 2019b). XIST influences progression of bladder cancer through suppressing p53 via binding to TET1 (Hu et al., 2019). Another route of participation of XIST in tumor growth and metastasis is exerted through regulation of miR-139-5p and Wnt/ β-catenin pathway (Hu et al., 2017).

In bladder cancer cells, XIST serves as a molecular sponge for miR-200c through which it enhances colony formation, self-renewal capacity and EMT in cancer stem cells -like cells (Xu et al., 2018). Another study has indicated parallel over-expressions of XIST and androgen receptor (AR) in bladder cancer cells. Mechanistically, XIST increases AR expression though sponging miR-124 (Xiong et al., 2017). Moreover, XIST can promote proliferation and metastatic ability of bladder cancer cells via modulating miR-139-5p expression and subsequent regulation of Wnt/β-catenin signaling pathway (Hu et al., 2017). Figure 5 depicts the interactions between XIST and miRNAs in bladder cancer cells.

### Glioma

**FIGURE 6:**
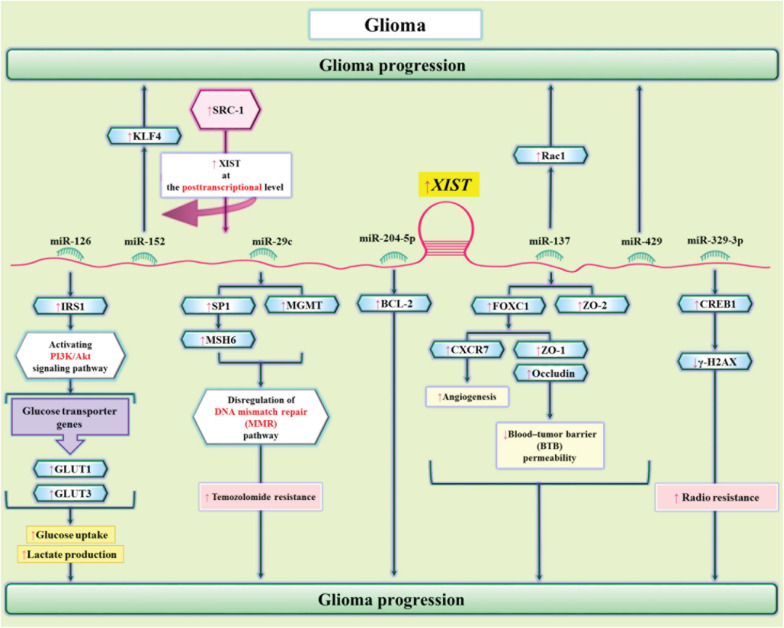
The interactions between XIST and miRNAs in glioma cells. XIST enhances glioma tumorigenic capacity and angiogenesis through sequestering miR-429 (Cheng et al., 2017). In addition, through sequestering miR-126, XIST regulates cell proliferation and glucose metabolism of glioma cells via influencing IRS1/PI3K/Akt axis (Cheng et al., 2020). The sponging effect of XIST on miR-29c modulates resistance of glioma cell to Temozolomide via DNA mismatch repair pathway (Du et al., 2017).

In glioma cells, XIST can modulate metabolism of glucose. XIST silencing has suppressed viability, migration, invasiveness, hypo-responsiveness to apoptotic stimuli, and glucose metabolism in glioblastoma. Mechanistically, XIST functions as a molecular sponge for miR-126 to subsequently regulate IRS1/PI3K/Akt pathway (Cheng et al., 2020). Another study in glioblastoma has shown the role of Steroid receptor coactivator-1 (SRC-1) in the regulation of XIST at posttranscriptional level. In fact, the impact of SRC-1 in enhancement of stemness features in glioblastoma is mediated through XIST. SRC-1 enhances expression of Kruppel-like factor 4 (KLF4) via the XIST/miR-152 axis (Gong et al., 2020). Moreover, miR-204-5p has been identified as another target of XIST in glioma cells. Interaction between XIST and miR-204-5p regulates expression of Bcl-2 (Shen et al., 2020). Figure 6 shows the interactions between XIST and miRNAs in glioma cells.

### Lung Cancer

**FIGURE 7:**
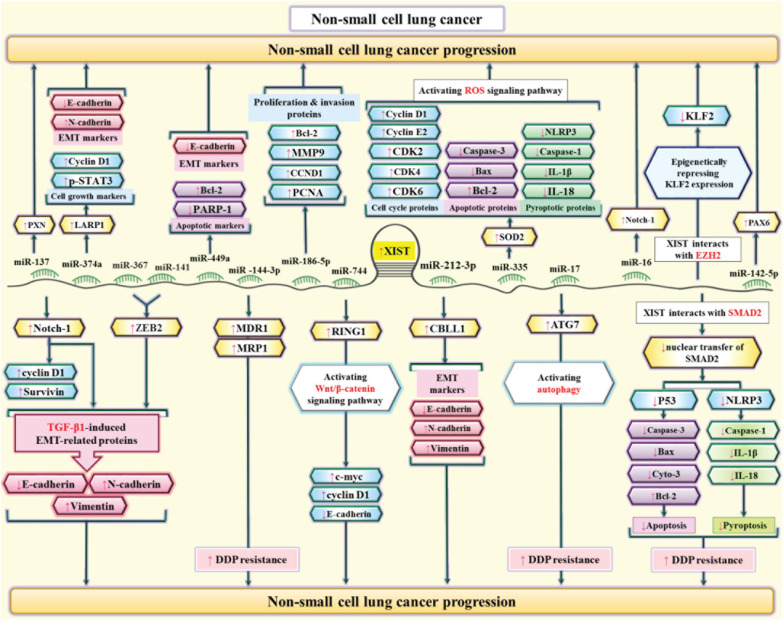
The interactions between XIST and miRNAs in lung cancer cells. XIST has an oncogenic role in lung cancer through different mechanisms including epigenetically silencing of KLF2 expression (Fang et al., 2016). Moreover, it can enhance viability and invasiveness of lung cancer cells through regulation of miR-137/PXN axis (Jiang et al., 2018). XIST can also enhance TGF-β-associated epithelial-mesenchymal transition through regulation of miR-367/141-ZEB2 (Li et al., 2018).

XIST has also been shown to be over-expressed in lung cancer cell lines promoting their proliferation ability through sponging miR-140. XIST silencing has repressed proliferation and enhanced apoptosis of lung cancer cells. Besides, inhibitor of apoptosis-stimulating protein of p53 (iASPP) has a prominent role in mediation of this effect (Tang et al., 2017). Expression of XIST expression has also been up-regulated in cisplatin-resistant lung cancer cells compared with the original cells. Up-regulation of this lncRNA has enhanced resistance to cisplatin through blocking apoptosis and increasing proliferation ability. These effects are mediated through sponging let-7i and regulating expression of BAG-1 (Sun et al., 2017). Figure 7 shows the interactions between XIST and miRNAs in lung cancer cells.

In addition to these types of malignancies, functional studies have verified the impact of XIST in the pathogenesis of almost all kinds of neoplasms. Supplementary Table 1 summarizes the results of *in vitro* studies.

## Animal Studies

In line with *in vitro* studies, abnormal expression of XIST affects tumorigenesis in animal models of cancer. Almost all studies have indicated that up-regulation of XIST enhances tumorigenic ability of cancer cells, while its silencing has the opposite effects (Table 1). However, XIST has a tumor suppressor role in animal models of oral squamous cell carcinoma and renal cell carcinoma. Most notably, animal studies in hepatocellular carcinoma, breast cancer, ovarian cancer and osteosarcoma have indicated inconsistent results regarding the role of XIST (Table 1).

**TABLE 1:** Outcomes of studies which evaluated function of XIST in animal models (Δ: knock down or deletion).

**Cancer type**	**Animal models**	**Results**	**References**
Bladder cancer	NOD/SCID mice	Δ XIST: ↓ tumorigenesis	Xu et al., 2018
	BALB/C nude mice	Δ XIST: ↓ tumorigenesis	Hu et al., 2017
	Nude mice	Δ XIST: ↓ tumorigenesis, ↑ PD sensitivity	Chen et al., 2020
Nasopharyngeal carcinoma	BALB/c nude mice	Δ XIST: ↓ tumorigenesis	Cheng et al., 2018
	BALB/c nude mice	Δ XIST: ↓ tumorigenesis	Zhao et al., 2020
	Nude mice	Δ XIST: ↓ tumorigenesis	Shi et al., 2020
Laryngeal squamous cell carcinoma	Nude mice	Δ XIST: ↓ tumorigenesis	Liu et al., 2020
Oral squamous cell carcinoma	Nude mice	↑ XIST: ↓ tumorigenesis	Li et al., 2020c
Esophageal squamous cell carcinoma	BABL/c nude mice	Δ XIST: ↓ tumorigenesis	Wu et al., 2017
Gastric cancer	BALB/c nude mice	Δ XIST: ↓ tumorigenesis	Li et al., 2020a
	BALB/c-nu/nu mice	Δ XIST: ↓ tumorigenesis, ↓ invasion	Ma et al., 2017
	BABL/c athymic nude mice	Δ XIST: ↓ tumorigenesis, ↓ metastasis	Chen et al., 2016
	BALB/c-nu/nu nude	Δ XIST: ↓ tumorigenesis	Li et al., 2020b
Colorectal cancer	BALB/C nude mice	Δ XIST: ↓ tumorigenesis	Sun et al., 2018
	BALB/c nude mice	Δ XIST: ↓ tumorigenesis	Wang et al., 2020b
	BALB/C nude mice	Δ XIST: ↓ tumorigenesis	Yang et al., 2020
	BALB/c nude mice	Δ XIST: ↑ anti-tumor effect of DOX, ↓ tumorigenesis	Zhuang et al., 2016
	Athymic nude mice	Δ XIST: ↓ tumorigenesis, ↓ metastasis	Chen et al., 2017
Pancreatic cancer	Nude mice	Δ XIST: ↓ tumorigenesis	Sun et al., 2018
	Nude mice	Δ XIST: ↓ tumorigenesis	Liu et al., 2020
	Nude mice	Δ XIST: ↓ tumorigenesis	Liang et al., 2017
Hepatocellular carcinoma	Nude mice	Δ XIST: ↓ tumorigenesis	Kong et al., 2018
	BALB/c-nu/nu mice	Δ XIST: ↓ tumorigenesis	Mo et al., 2017
	BALB/c thymus-free nude mice	↑ XIST: ↓ tumorigenesis	Zhang et al., 2019
	Nude mice	↑ XIST: ↓ tumorigenesis	Lin et al., 2018
Renal cell carcinoma	BALB/C mice	↑ XIST: ↓ tumorigenesis	Sun et al., 2019
Lung cancer	BALB/c nude mice	↑ XIST: ↑ tumorigenesis, ↑ cisplatin resistance	Sun et al., 2017
Non-small cell lung cancer	BABL/c athymic nude mice	Δ XIST: ↓ tumorigenesis	Jiang et al., 2018
	Nude mice	Δ XIST: ↓ tumorigenesis	Qiu et al., 2019
	BALB/c nude male mice	Δ XIST: ↓ tumorigenesis, ↑ DDP chemosensitivity	Xu et al., 2020
	BALB/c nude mice	Δ XIST: ↓ tumorigenesis	Zhang et al., 2017
	Nude mice	Δ XIST: ↓ tumorigenesis	Zhou et al., 2019c
	BALB/c nude mice	Δ XIST: ↓ tumorigenesis, ↓ liver metastasis	Wang et al., 2019
	BALB/c nude mice	Δ XIST: ↓ tumorigenesis	Fang et al., 2016
	BALB/c nude mice	Δ XIST: ↓ tumorigenesis, ↓ DDP chemoresistance	Tian et al., 2019
	BALB/c nude mice	Δ XIST: ↓ tumorigenesis	Jiang et al., 2020
	Nude mice	Δ XIST: ↓ pulmonary metastasis	Li et al., 2018
	Nude mice	Δ XIST: ↓ tumorigenesis	Wang et al., 2017
Breast cancer	BALB/c nude mice	↑ XIST: ↓ tumorigenesis	Li et al., 2020d
	Nude mice	Δ XIST: ↑ tumorigenesis, ↑ brain metastasis, ↑EMT, ↑ stemness	Xing et al., 2018
	BALB/c nu/nu mice	Δ XIST: ↑ tumorigenesis, ↑ migration	Zhao et al., 2020
	BALB/c nude mice	↑ XIST: ↓ tumorigenesis	Liu et al., 2020
Ovarian cancer	Athymic nude mice	↑ XIST: ↓ tumorigenesis, ↑ cisplatin chemosensitivity	Wang et al., 2018
	Nude mice	↑ XIST: ↓ tumorigenesis, ↑ paclitaxel sensitivity, ↓ CD44 + /CD24-population cells	Huang et al., 2020
	BALB/c mice	↑ XIST: ↓ tumorigenesis	Guo et al., 2021
Prostate cancer	BALB/C nude mice	↑ XIST: ↓ tumorigenesis	Du et al., 2017
Osteosarcoma	BALB/c nude mice	↑ XIST: ↓ tumorigenesis	Zhang and Xia, 2017
	BALB/C nude mice	Δ XIST: ↓ tumorigenesis	Xu et al., 2017
	BALB/c athymic nude mice	Δ XIST: ↓ tumorigenesis	Gao et al., 2019
	BALB/c nude mice	Δ XIST: ↓ tumorigenesis, ↓ metastasis	Yang et al., 2018
Glioma	BALB/c nude mice	Δ XIST: ↓ tumorigenesis	Cheng et al., 2020
	BALB/c nude mice	Δ XIST: ↓ tumorigenesis, ↑ survival time	Shen et al., 2020
	BALB/c nude mice	Δ XIST: ↓ tumorigenesis, ↓ angiogenesis	Cheng et al., 2017
	BALB/C athymic nude mice	Δ XIST: ↓ tumorigenesis, ↑ survival time	Yao et al., 2015
	Nude mice	Δ XIST: ↓ tumorigenesis	Wang et al., 2020c
Thyroid cancer	Athymic nude mice	Δ XIST: ↓ tumorigenesis	Liu et al., 2018
Acute myeloid leukemia	BALB/c nude mice	Δ XIST: ↓ tumorigenesis	Wang et al., 2020a
Retinoblastoma	BALB/c nude mice	Δ XIST: ↓ tumorigenesis, ↑ VCR sensitivity	Yao et al., 2020
Cervical cancer	Athymic BALB/c mice	Δ XIST: ↓ tumorigenesis, ↓ EMT	Chen et al., 2019
	Nude mice	Δ XIST: ↓ tumorigenesis	Liu et al., 2020
Neuroblastoma	BALB/c nude mice	Δ XIST: ↓ tumorigenesis, ↑ survival time	Zhang et al., 2019
	BALB/c nude mice	Δ XIST: ↓ tumorigenesis	Yang et al., 2020
Chordoma	Nude mice	Δ XIST: ↓ tumorigenesis, ↑ apoptosis	Hai et al., 2020

## Human Studies

Experiments in clinical samples obtained from patients have shown that expression of XIST is principally increased in tumoral samples compared with nearby non-cancerous samples (Supplementary Table 2). However, in oral squamous cell carcinoma, its expression has been decreased (Li et al., 2020c). In hepatocellular carcinoma, most of studies have indicated its down-regulation (Chang et al., 2017; Lin et al., 2018; Zhang et al., 2019). However, few studies have reported opposite results (Mo et al., 2017; Kong et al., 2018). Similarly, different studies in breast cancer, ovarian cancer, osteosarcoma and renal cell carcinoma(Supplementary Table 2) have reported inconsistent results. Moreover, expression levels of XIST have been correlated with patients’ survival in different kinds of cancers including bladder cancer, esophageal squamous cell carcinoma, nasopharyngeal carcinoma, lung cancer, gastric cancer, colorectal cancer and breast cancer.

In nasopharyngeal carcinoma, XIST expression levels could differentiate tumoral tissues from nearby non-cancerous samples with diagnostic power of 0.813 (Song et al., 2016). In colorectal cancer, up-regulation of XIST in extracellular vesicles isolated from serum samples had an appropriate diagnostic value [Area under curve (AUC) = 0.86, sensitivity = 0.88 and specificity = 0.90]. Most notably, over-expression of XIST in serum extracellular vesicles has been associated with survival rates (Yu et al., 2020). Expression levels of XIST have also been shown to be appropriate markers for follow-up of patients with lung cancer, since they have been reduced following surgical removal of tumors. Receiver operating characteristic curves have demonstrated the ability of XIST expression levels in separation between the patients and healthy controls with an AUC value of 0.834. In addition, combination of expression levels of XIST and HIF1A-AS1 in serum samples has enhanced the diagnostic power (Tantai et al., 2015). Finally, serum levels of XIST could separate breast cancer patients from healthy controls with AUC value of 0.78 (Zhao et al., 2018). Table 2 summarizes the outcomes of studies which evaluated this aspect of XIST application in clinical settings.

**TABLE 2:** Diagnostic value of XIST in cancers.

**Cancer type**	**Numbers of clinical samples**	**Distinguish between**	**Area under curve**	**Sensitivity (%)**	**Specificity (%)**	**References**
Nasopharyngeal carcinoma (NPC)	108 pairs of NPC tissues and ANTs	NPC patients vs. controls	0.813	0.886	0.795	Song et al., 2016
Early gastric cancer (EGC)	76 pairs of EGC tissues and ANTs and EGC plasma	EGC patients vs. controls	0.733	0.846	0.590	Lu et al., 2017
Colorectal cancer	120 serum specimens from CRC responding and non-responding patients to 5FU treatment	CRC patients showing response to 5FU treatment vs. patients showing no response	0.717	0.756	0.683	Xiao et al., 2017
	Serum EVs from 94 CRC patients and 41 healthy participants	CRC patients vs. healthy controls	0.864	0.883	0.902	Yu et al., 2020
Non-small cell lung cancer	32 pairs of NSCLC tumor tissues and ANTs 64 serum samples	NSCLC vs. controls	0.834	0.726	0.935	Tantai et al., 2015
Breast cancer	36 serum samples from breast cancer patients and 32 control healthy subjects	Breast cancer patients vs. healthy controls	0.78	0.67	0.89	Zhao et al., 2018
Thyroid cancer	77 pairs of thyroid cancer tissue samples and ANTs	Thyroid cancer patients vs. healthy controls	0.7360	–	–	Liu et al., 2018

## Discussion

Although XIST has been primarily identified as a transcript whichregulates X inactivation, subsequent studies have show thatthislncRNA has several regulatory roles beyond thisphysiologicalprocess. In addition to interacting with chromatin modifyingmolecules, XIST can be served as a molecularsponge for miRNAs to modulate expression of miRNA targets. miR-362-5p/UBAP1, miR-125b-5p/NLRC5, miR-200c-3p/ANLN, miR-337/JAK2, miR-132/PXN, miR-497/MACC1, miR-185/TGF-β1, miR-497-5p/FOXK1, miR-141-5p/TGF-β2, miR-152/KLF4, and let-7i/BAG-1 are among molecular cascades downstream of XIST which are involved in the carcinogenesis process.

XIST can modulate resistance to chemotherapeutic agents in a number of cancers including breast and lung cancers (Sun et al., 2017; Zhang et al., 2020). Thus, modulation of its expression might beregarded as a strategy for combatting chemoresistance of cancercells. However, tissue-specific effects of XIST in conferring resistance to chemotherapeutic agents should be considered. Most of the above-mentioned studies have indicated an oncogenic role for XIST. However, in prostate cancer, a single study has indicated a tumor suppressor role for this lncRNA (Du et al., 2017). Similar result has been reported for XIST in oral squamous cell carcinoma (Li et al., 2020c). In hepatocellular carcinoma, breast cancer, ovarian cancer, osteosarcoma and renal cell carcinoma, different studies have reported inconsistent results (Supplementary Table 2). Most notably, animal studies in hepatocellular carcinoma, breast cancer, ovarian cancer and osteosarcoma have indicated inconsistent results regarding the role of XIST. Although these discrepancies might be due to possible tissue-specific roles for XIST or differences in cell lines (particularly passage number) and animal models, future studies with larger sample sizes from different ethnic groups are needed to solve these discrepancies.

XIST has both diagnostic and prognostic values in different cancers, albeit the prognostic value of this lncRNA has been more validated. Both tissue and serum levels of XIST can be used to distinguish disease status, yet the latter source is superior regarding the non-invasive route of access. The best diagnostic power values have been reported in CRC, NSCLC, and nasopharyngeal carcinoma, respectively. However, all of these studies lack validation in independent samples. So, future studies should assess this aspect of XIST application in larger cohorts of patients.

In brief, XIST has been shown to affect carcinogenic process possibly in a tissue-specific manner. Therefore, therapeutic strategies targeting this lncRNA should consider this point to design a personalized regimen for treatment of cancer.

## Author Contributions

MT and SG-F wrote the draft and revised it. SD, MF, and SM collected the data and designed the tables and figures. All the authors approved submitted version.

## Conflict of Interest

The authors declare that the research was conducted in the absence of any commercial or financial relationships that could be construed as a potential conflict of interest.
