# Supplementary material for: X-Inactive-Specific Transcript: Review of Its Functions in the Carcinogenesis
Source: Front Cell Dev Biol. 2021 Jun 11;9:690522. doi: 10.3389/fcell.2021.690522 (PMC8226258; doi:10.3389/fcell.2021.690522)
Supplement: Supplementary file 1 [file Table_1.docx]

**Supplementary Table S1** - Brief results of investigations which assessed expression of XIST in cell lines (∆: knock-down, DDP: Cisplatin (cis-diamminedichloroplatinum (II)), TMZ: Temozolomide, MMR: Mismatch repair, HBMVECs: Human brain microvascular endothelial cells, PD: Platycodin D, BCSC: Bladder cancer stem cell, VCR: Vincristine, BTB: blood-tumor barrier, ROS: reactive oxygen species).

| Cancer type | Targets/ Regulators and Signaling Pathways | Cell line | Function | Reference |
| --- | --- | --- | --- | --- |
| Bladder cancer (BC) | miR-200c, CD133, CD44, KLF4, OCT-4, ABCG2, E-cadherin, vimentin, ZEB1, ZEB2 | 5637, T24 | ∆ XIST: ↓ cell clone formation, ↓ self-renewal, ↓ EMT in BCSC-like cells | (31) |
|  | miR‑124, AR, c-myc, p27, MMP13, MMP9 | TCC-SUP, EJ, SW780, UM-UC-3, SV-HUC-1 | ∆ XIST: ↓ cell proliferation, ↓ migration, ↓ invasion | (32) |
|  | miR‑139‑5p, Wnt1, β-catenin, cyclin-D1, Wnt/β-catenin | SV-HUC-1, T24, 5637, EJ, UMUC3, J82, HEK-293T | ∆ XIST: ↓ cell proliferation, ↑ cell cycle arrest, ↓ metastasis, ↓ invasion, ↑apoptosis | (33) |
|  | miR-335 | T24, RT4 | ∆ XIST: ↓ cell proliferation, ↓ colony formation, ↓ invasion, ↓ migration, ↑apoptosis, ↑ PD sensitivity | (46) |
|  | p53, TET1 | 5637, T24 | ∆ XIST: ↓ cell proliferation, ↓ migration, ↑apoptosis, ↑ G0/G1 cell cycle arrest | (34) |
|  | miR‑133a | SV‑HUC‑1, T24, 253J, RT112, HT‑1376 | ∆ XIST: ↓ cell proliferation, ↓ migration | (35) |
| Nasopharyngeal carcinoma (NPC) | miR-34a-5p, E2F3 | UNE-1, CNE-1, HNE-1, CNE-2, C666-1, HONE-1 | ∆ XIST: ↓ cell proliferation | (47) |
|  | miR-491-5p | NPC cell lines CNE1, CNE2, SUNE-1, C666-1 | ∆ XIST: ↓ cell proliferation , ↓ invasion, ↓ migration, ↑ apoptosis | (48) |
|  | miR-381-3p, NEK5 | HK-1, C666-1, NP69 | ∆ XIST: ↓ hypoxia-induced glycolysis, ↓ Migration, ↓ Invasion | (49) |
|  | miR-29c | CNE-1, CNE-2 | ∆ XIST: ↓ cell proliferation, ↑ radiosensitivity, ↓ DNA damage repair | (50) |
|  | miR-148a-3p, ADAM17 | UNE-1, CNE-1, CNE-2, C666-1, HNE1 | ∆ XIST: ↓ cell proliferation , ↓ invasion, ↓ migration, ↓ EMT, ↑ apoptosis | (51) |
| Laryngeal squamous cell carcinoma (LSCC) | miR-125b-5p, TRIB2 | AMC-HN-8, M4E, NP69 | ∆ XIST: ↓ cell proliferation, ↓ migration, ↓invasion, ↑ apoptosis | (52) |
|  | miR-124-3p, EZH2 | Hep-2, HEK293T | ∆ XIST: ↓ cell proliferation, ↓ migration, ↓invasion | (53) |
| Oral squamous cell carcinoma (OSCC) | miR-455-3p, BTG2 | CAL27, SCC15, SCC25, TSCCA | ↑ XIST: ↓ cell proliferation, ↓ migration, ↓ invasion | (54) |
| Esophageal squamous cell carcinoma (ESCC) | miR-494, CDK6 , JAK2/STAT3 signal pathway | HEEC, SKGT-4, Bic-1, TE-1, HCE-4 , HCE-7 | ∆ XIST: ↓ cell proliferation, ↓ invasion, ↓ migration, ↑ apoptosis | (55) |
|  | miR-101, EZH2 | KYSE30, KYSE510, KYSE410, KYSE520, KYSE140, KYSE150 | ∆ XIST: ↓ cell proliferation, ↓ invasion, ↓ migration | (56) |
| Gastric cancer (GC) | miR-337, JAK2 | GES-1, HGC-27, AGS, HEK-293T | ∆ XIST: ↓ cell proliferation, ↓ invasion, ↓ migration | (11) |
|  | miR -132, PXN | BGC-823, SGC7901, MKN45, MKN28, AGS, and HGC-27, GES-1 | ∆ XIST: ↓ tumor formation ability, ↓ cell proliferation, ↓ migration, ↑ apoptosis | (12) |
|  | miR-497, MACC1 | SGC-7901, BGC-823, HGC-27 and MKN-28, GES-1 | ∆ XIST: ↓ cell proliferation, ↓ invasion, ↓ EMT, ↓ G1/S cell cycle progression, ↑ apoptosis | (13) |
|  | miR-185, TGF-β1 | MGC803, BGC823, GES-1, SGC-7901, AGS, KATOIII, HEK-293T | ∆ XIST: ↓ cell proliferation, ↓ migration, ↓ invasion | (14) |
|  | miR-101, EZH2 | SGC7901, HGC27, BGC823, MKN45, MKN28, AGS, GES-1 | ∆ XIST: ↓ cell proliferation, ↓ invasion, ↓ migration | (16) |
|  | EPHA1, HNF4A, NFκB pathway | HS 746T, NCI-N87, GES-1 | ∆ XIST: ↓ cell proliferation, ↓ invasion | (57) |
| Colorectal cancer | miR-34a, WNT1, c-Myc, cyclinD1, MMP-7, Wnt/β-catenin signaling pathway | Lovo, SW480, HCT116 and HT29, NCM460 | ∆ XIST: ↓ cell proliferation, ↓ invasion | (17) |
|  | Thymidylate synthase (TS) | HT29, HCT116, FHC, HT29, 5FU-Resistant (5FU-R) CRC cell lines: HT29 5FU-R, HCT116 5FU-R | ∆ XIST: ↓ cell viability, ↓ 5FU resistance | (18) |
|  | miR‑497‑5p, FOXK1 | LOVO, SW480, HT-29, HCT-116, FHC | ∆ XIST: ↓ cell proliferation, ↓ migration, ↓ invasion | (19) |
|  | miR-93-5p, HIF-1A/AXL signaling pathway | W480, LoVo, NCM460 | ∆ XIST: ↓ cell proliferation, ↓ migration, ↓ EMT, ↑ apoptosis | (58) |
|  | miR-124, SGK1 | HCT116, LoVo, DOX-resistant CRC cells: HCT116/DOX, LoVo/DOX | ∆ XIST: ↓ Doxorubicin resistance, ↑ apoptosis | (59) |
|  | miR‐486‐5p, NRP‐2 | HCT116, HT29, SW620, SW480, CCD-116CoN | ∆ XIST: ↓ cell proliferation, ↓ EMT, ↓ migration, ↓ invasion, ↑ apoptosis | (21) |
|  | miR-137, EZH2 | LoVo, HT29, SW620 | ∆ XIST: ↓ migration, ↓ invasion, ↓ EMT | (22) |
|  | miR-30a-5p, ROR1 | W480, HCT116, Lovo, SW620, HEK293T | ∆ XIST: ↓ cell proliferation, ↑ apoptosis, ↑ chemosensitivity | (60) |
|  | miR-132-3p, MAPK1 | LOVO, HCT116, SW620, SW480, HT29, NCM460, 293T | ∆ XIST: ↓ cell proliferation, ↑ G0/G1 cell cycle arrest | (23) |
|  | miR-200b-3p, ZEB1 | HCT116, HT-29, SW620, SW480, DLD-1, RKO, LoVo, CCD-116CoN, HEK-293T | ∆ XIST: ↓ cell proliferation, ↓ invasion, ↓ migration, ↓ EMT, ↓ CRC stem cell formation | (20) |
| Pancreatic cancer | miR-34a-5p | PANC-1, ASPC-1, MIA PaCa-2, HPAC, CFAPC-1 and BxPC-3, HPDE, 293T | ∆ XIST: ↓ cell proliferation, ↓ migration, ↓ invasion, ↑ apoptosis | (24) |
|  | miR‑137, Notch1 | H6c7, Patu8988, SW1990, MIA PaCa-2, AsPC-1, CFPAC-1, BxPC-3, Panc-1 | ∆ XIST: ↓ cell proliferation | (25) |
|  | miR-141-3p, TGF-β2 | PANC-1, HEK293T | ∆ XIST: ↓ cell proliferation, ↓ migration, ↓ invasion | (26) |
|  | miR-429, ZEB1 | ASPC-1, PANC-1, HPAC, BxPC-3, CFPAC-1, HPDE | ∆ XIST: ↓ migration, ↓ invasion, ↓ EMT | (28) |
|  | miR-140, miR-124, iASPP, CDK1, P21 | CS-PE, AsPC-1, BxPC3, SW1990, PANC-1, CAPAN-1, CAPAN-2 | ∆ XIST: ↓ cell proliferation, ↑ G0/G1 cell cycle arrest | (27) |
|  | miR-34a,YAP, EGFR | AsPC-1, MIAPaCa-2, PATU8988T, PANC-1 , SW1990, HPDE6-C7 | ∆ XIST: ↓ cell proliferation, ↓ migration, ↓ invasion, ↓ TGF-β1-induced EMT, ↑ apoptosis | (30) |
|  | miR-133a, EGFR, Akt signaling | H6c7, Patu8988, SW1990, BxPC-3, AsPC-1, CFPAC-1, Panc-1 | ∆ XIST: ↓ cell proliferation | (29) |
| Hepatocellular carcinoma (HCC) | miR-194-5p, MAPK1 | MHCC97L, MHCC97H, HepG2, SMMC7221, Huh7, Bel-7402, HL-7702 | ∆ XIST: ↓ migration, ↓ invasion | (61) |
|  | miR-181a, PTEN | HCCLM3, HepG2, Hep3B, SMMC-7721, Huh7, HL-7702, L-02 | ∆ XIST: ↑ cell proliferation, ↑ migration, ↑ invasion | (62) |
|  | miR-139-5p, PDK1, AKT signaling | LM9, Hh7, Hep3B, and HepG2, LO2 | ∆ XIST: ↓ cell proliferation, ↑ apoptosis | (63) |
|  | miR-497-5p, PDCD4 | HepG2, HepB3, Huh7, SMMC-7721, MHCC-97L , Bel-7402, LO2, HEK293T | ↑ XIST: ↓ cell proliferation, ↓ migration | (64) |
|  | miR-155-5p, JPX, SOX6, PTEN | HepG2 | ↑ XIST: ↓ cell proliferation, ↑ cisplatin sensitivity, ↑ apoptosis | (65) |
|  | miR -92b, Smad7 | QSG-7701, SMMC-7721, Huh-6, Huh-7, HepG2, HCCLM3, QGY-7703 | ↑ XIST: ↓ cell proliferation, ↓ metastasis | (66) |
| Renal cell carcinoma (RCC) | miR-106b-5p, P21 | ACHN, Caki-1, Caki-2, 786-O, HK2 | ↑ XIST: ↓ cell proliferation, ↑ G0/G1 cell cycle arrest | (67) |
|  | miR-302c, SDC1 | 786-O, Caki-1, ACHN, HK-2, HEK293T | ∆ XIST: ↓ cell proliferation, ↑apoptosis | (68) |
| Lung cancer | miR-140, iASPP | A549, H1299 | ∆ XIST: ↓ cell proliferation, ↓ metastasis, ↑apoptosis | (41) |
|  | let-7i, BAG-1 | A549, A549/DDP | ∆ XIST: ↓ cell proliferation, ↓ cisplatin resistance, ↑ apoptosis | (42) |
| Non-small cell lung cancer (NSCLC) | miR-137, Notch-1 pathway | A549, H358, H460, H1299, PC9, 16HBE | ∆ XIST: ↓ cell proliferation, ↓ TGF-β1-induced EMT | (69) |
|  | miR-137, PXN | A549, H450, 95D, H1299, SPC-A-1, H522, 16-HBE | ∆ XIST: ↓ cell viability, ↓ invasion | (44) |
|  | miR-374a, LARP1 | H520, A549, H1299, H1975 | ∆ XIST: ↓ cell proliferation, ↓ invasion, ↓ migration | (70) |
|  | miR-212-3p, CBLL1 | H522, Calu3, 16HBE | ∆ XIST: ↓ cell proliferation, ↓ invasion, ↓ migration, ↓ EMT | (71) |
|  | SMAD2, p53, NLRP3 | A549, H1299, A549/DDP, H1299/DDP | ∆ XIST: ↓ cell proliferation, ↓ colony formation, ↑ apoptosis, ↑ pyroptosis, ↑ DDP chemosensitivity | (72) |
|  | miR-449a, E-cadherin, Bcl-2 | NL9980, NCI-H1299, NCI-H460, SPC-A-1, A549, BEAS-2B | ∆ XIST: ↓ cell proliferation, ↓ invasion, ↓ migration, ↑ apoptosis | (73) |
|  | miR-16, CDK8 | A549, H1299, H292, H460 | ∆ XIST: ↓ cell proliferation, ↓ colony formation, ↓ migration, ↑ G0/G1 cell cycle arrest | (74) |
|  | miR-744, RING1, Wnt/β-catenin pathway | A549, H1299, H23, H522, H460, H1650, 95D | ∆ XIST: ↓ cell proliferation, ↓ invasion, ↓ migration | (75) |
|  | EZH2, KLF2 | A549, SK-MES-1, H1299, 95D, H460, H520, H1975, H157, SK-LU-1, and SPC-A-1, 16HBE | ∆ XIST: ↓ cell proliferation, ↓ invasion, ↓ migration | (43) |
|  | miR -144-3p, MDR1, MRP1 | H460/DDP, A549/DDP | ∆ XIST: ↓ cell survival, ↓ cell proliferation, ↓ migration, ↑ apoptosis | (76) |
|  | miR-142-5p, PAX6 | 16HBE, A549, H292, H460, H1299 | ∆ XIST: ↓ cell proliferation, ↓ migration, ↓ invasion, ↑ apoptosis | (77) |
|  | miR-367, miR-141, ZEB2 | A549, H226 | ∆ XIST: ↓ TGF-β-induced EMT, ↓ migration, ↓ invasion | (45) |
|  | miR-186-5p | A549, H1299, SK-MES-1, Calu-3 | ∆ XIST: ↓ cell proliferation, ↓ invasion, ↑ apoptosis | (78) |
|  | miR-335, SOD2, ROS signal pathway | A549, H1299, SK-MES-1, Calu-3, HBE | ∆ XIST: ↓ cell proliferation, ↑ apoptosis, ↑ ROS generation, ↑ pyroptosis | (79) |
|  | miR-17, ATG7 | A549, H1299, cisplatin-resistant DDP A549 | ∆ XIST: ↓ cell proliferation, ↑ chemosensitivity to cisplatin, ↓ autophagy | (80) |
| Breast cancer | miR-125b-5p , NLRC5 | MCF-7, MDA-MB-231, SKBR3, MCF-10A | ∆ XIST: ↓ cell proliferation, ↓ migration, ↓ invasion, ↑ apoptosis | (9) |
|  | miR-200c-3p, ANLN | MDA-MB-231, doxorubicin resistant cells: MDA-MB-231/ADM | ∆ XIST: ↓ cell proliferation, ↑apoptosis, ↓ doxorubicin resistance | (10) |
|  | miR-454 | MDA-MB-468, MDAMB-231 | ↑ XIST: ↓ cell proliferation, ↓ EMT, ↑ apoptosis | (8) |
|  | miRNA-503, MSN, c-Met | MCF7, ZR75-1, SKBR3, MDA-MB-231 | ↑ XIST: ↓ cell proliferation, ↓ migration, ↓ brain metastasis | (81) |
|  | miR-155, CDX1 | MCF-7, ZR-75-1, HCC-1937, MDA-MB-231, MDA-MB-468, MDA-MB-453 | ↑ XIST: ↓ cell proliferation, ↓ invasion, ↓ migration | (82) |
|  | pAKT, AKT pathway, Jpx, PHLPP1, HDAC3, SPEN | MCF-7, MDA-MB-468, Hs578T, MDA-MB-231, M10 | ∆ XIST: ↑ cell viability | (83) |
|  | miR-20a | MCF-12A, BT-474, BT-483, AU565, HCC1806 | ∆ XIST: ↑cell proliferation, ↑ migration, ↑ invasion | (84) |
|  | miR-101, C/EBPα, KLF6 | MCF-7, OVCAR3, HEK293T | ∆ XIST: ↑ M1-to-M2 conversion (macrophage polarization to M2 phenotype), ↑cell proliferation, ↑ migration | (85) |
|  | miR-362-5p, UBAP1 | CCD-1095SK, MCF7 , MDA-MB-231 | ↑ XIST: ↓ cell proliferation, ↓ migration, ↓ invasion, ↑ apoptosis | (7) |
| Ovarian cancer (OC) | has-miR-214-3p | CAOV3, OVCAR3 | ↑ XIST: ↓ cell proliferation, ↓ invasion, ↑ cisplatin chemosensitivity | (86) |
|  | - | OVCAR3, OV90, A2780, SKOV3, HOSE | ∆ XIST: ↑cell proliferation, ↑ migration, ↑ invasion | (87) |
|  | miR-93-5p, KMT2C | SKOV3, ES-2, TOV21G, RMG-1 | ↑ XIST: ↑ paclitaxel sensitivity, ↓ cancer stem cell population | (88) |
|  | miR-106a | A2780, SKOV3, OV90, CAOV3, OVCAR3 | ↑ XIST: ↓ cell proliferation, ↑ apoptosis | (89) |
| Prostate cancer (PCa) | miR-23a, RKIP | PC3, DU145, LNCAP, RWPE1 | ↑ XIST: ↓ cell proliferation, ↓ invasion, ↓ migration | (90) |
| Osteosarcoma (OS) | - | HOS, SAOS-2, SOSP-9607, MG63, U2-OS, hFOB 1.19 | ∆ XIST: ↓ cell proliferation | (91) |
|  | miR‑137, MMP2, MMP9 | Saos‑2, U2OS, HOS, MG63, HFOB1.19 | ∆ XIST: ↓ cell proliferation, ↓ invasion | (92) |
|  | miR-21-5p, PDCD4 | MG63, U2OS, Saos2, HOS, SOSP-9697, SV40, hFOB 1.19 | ↑ XIST: ↓ cell proliferation, ↓ invasion, ↓ migration, ↓ EMT, ↑ apoptosis | (93) |
|  | miR-320b, RAP2B | U2OS, Saos-2, MG63, MNNG/HOS, HFOB 1.19 | ∆ XIST: ↓ cell proliferation, ↓ invasion | (94) |
|  | NF-kB, PUMA, p65, bax | U2OS | ∆ XIST: ↓ cell proliferation, ↑ apoptosis | (95) |
|  | miR-195-5p, YAP | MG-63, 143B, MHM, SJSA1 | ∆ XIST: ↓ cell proliferation, ↓ invasion, ↓ EMT | (96) |
|  | miR-375-3p, AKT/mTOR signaling pathway | MG-63, HOS, U2-OS, Saos-2, hFOB1.19 | ∆ XIST: ↓ cell proliferation, ↓ autophagy, ↑ apoptosis | (97) |
|  | - | KHOS‑240S, SaOS2, MG‑63, SOSP‑9607, U2OS, hFOB1.19 | ∆ XIST: ↓ cell proliferation, ↓ migration, ↓ invasion, ↑ apoptosis, ↑ cell cycle arrest | (98) |
|  | miR-153, SNAI1  FBN, Snail, Vimentin, E-cadherin | U2OS, SaoS-2, HOS, MG63, 143B, hFOB 1.19 | ∆ XIST: ↓ H2O2-induced migration, ↓ invasion, ↓ EMT | (99) |
|  | P21, EZH2 | U2OS, Saos-2, 143B, MG- 63, HOS | ∆ XIST: ↓ cell proliferation, ↑ G0/G1 cell cycle arrest, ↑ apoptosis | (100) |
| Glioma | miR-126, IRS1/PI3K/Akt pathway | U87MG, U251, U343, Hs683, LN215, A172, HA1800 | ∆ XIST: ↓ cell viability, ↓ migration, ↓ invasion, ↑ apoptosis, ↓ glucose metabolism, ↓ lactate production | (36) |
|  | miR-152, KLF4 | LN229, U251, U87-MG, T98G, U118, C6, SVG-p12, HEK293T, HeLa | ∆ XIST: ↓ stemness | (37) |
|  | miR-204-5p, Bcl-2 | U87, U251 | ∆ XIST: ↓ cell proliferation, ↓ migration, ↓ invasion, ↑ apoptosis | (38) |
|  | miR-29c, SP1, MGMT, MSH6, MMR pathway | U251, U373, LN229, U118, LN229, LN229/TMZ, U251/TMZ | ∆ XIST: ↓ cell proliferation, ↓ TMZ resistance | (40) |
|  | miR-429 | A1800, A172, U251, HBMVECs | ∆ XIST: ↓ cell proliferation, ↓ migration, ↓ angiogenesis, ↑ frequency of G1/ S phase cells, ↓ G2/M phase cells | (39) |
|  | miR-137, Rac1 | U251, LN229 | ∆ XIST: ↓ cell proliferation, ↑ G1/G0 cell cycle arrest | (101) |
|  | miR-152 | HEK- 293T, GSCs | ∆ XIST: ↓ cell proliferation, ↓ migration, ↓ invasion, ↑ apoptosis | (102) |
|  | miR-329-3p, CREB1 | U251, A172 | ∆ XIST: ↓ cell proliferation, ↓ invasion, ↑ apoptosis, ↑sensitivity to X-ray radiation | (103) |
|  | miR-137, FOXC1, ZO-2, CXCR7 | hCMEC/D3, U87MG, U118MG, HEK293T | ∆ XIST: ↓ cell proliferation, ↓ migration , ↑ BTB permeability, ↓ angiogenesis | (104) |
| Thyroid cancer | miR-34a, MET-PI3K-AKT signaling | FTC133, BCPAP, TEC, TPC-1 | ∆ XIST: ↓ cell proliferation | (105) |
| Papillary thyroid carcinoma (PTC) | miR‑101‑3p, CLDN1 | NPA87, TPC-1, KAT-5, HT-ori 3-1 | ∆ XIST: ↓ migration, ↓ invasion | (106) |
|  | miR-141 | TPC-1, HTH83, 8505C, SW1736 | ∆ XIST: ↓ cell proliferation, ↓ migration, ↓ invasion | (107) |
| Acute myeloid leukemia (AML) | miR‑29a, MYC | KG-1 | ∆ XIST: ↓ cell proliferation, ↑ apoptosis, ↑ Adriamycin sensitivity | (108) |
| Retinoblastoma (RB) | miR-124, STAT3 | Y79, Weri-Rb1, SO-RB-50, HXORB44, ARPE-19 | ∆ XIST: ↓ cell proliferation, ↑ G1/G0 cell cycle arrest, ↑ apoptosis | (109) |
|  | miR-140-5p, SOX4 | ARPE-19, Y79, Weri-Rb1, SO-Rb50, HXO-RB44 | ∆ XIST: ↓ cell proliferation, ↓ invasion | (110) |
|  | miR-101, ZEB1, ZEB2 | SO-RB50, Y79, and Weri-Rb1, ARPE-19 | ∆ XIST: ↓ cell proliferation, ↓ migration, ↓ invasion, ↑ apoptosis | (111) |
|  | miR-204-5p | WERI-RB1, Y79, ARPE-19 | ∆ XIST: ↓ cell proliferation, ↓ autophagy, ↑ VCR sensitivity, ↑ apoptosis | (112) |
|  | NKILA | WERI-Rb-1, Y79 | ↑ XIST: ↑ cell proliferation,↑ migration, ↑ invasion | (113) |
| Cervical cancer (CC) | MiR-140-5p, ORC1 | CaSki, HeLa, C33A, SiHa, 293T | ∆ XIST: ↓ cell proliferation, ↓ cell cycle, ↑ apoptosis | (114) |
|  | miR-889-3p, SIX1 | Ect1/E6E7, Me180, Hela | ∆ XIST: ↓ cell proliferation, ↓ invasion, ↓migration, ↑ apoptosis | (115) |
|  | miR-200a, Fus | GH329, Hela, Caski, C4-1, Siha | ∆ XIST: ↓ cell proliferation, ↓ invasion, ↓ EMT, ↑ apoptosis | (116) |
| Pituitary neuroendocrine tumor (PitNET) | miR -424-5p, bFGF | Invasive PitNETcells | ∆ XIST: ↓ cell proliferation, ↓ migration, ↓ invasion, ↑ cell cycle arrest, ↑apoptosis | (117) |
| Malignant melanoma (MM) | miR-21, PI3KR1, Bax, Bcl-2, PI3K/AKT signaling pathway | A375, SK-MEL-28, WM451, MNT-1, HEM-l | ∆ XIST: ↓ cell proliferation, ↓ migration, ↑ oxaliplatin sensitivity | (118) |
| Neuroblastoma (NB) | DKK1, EZH2 | SH-SY5Y, SK-N-BE(2), HEK293 | ∆ XIST: ↓ cell proliferation, ↓ migration, ↓ invasion | (119) |
|  | miR‑375, L1CAM | SK-N-AS, IMR-32, Kelly, HUVEC | ∆ XIST: ↓ cell proliferation, ↓ cell cycle progression, ↑ radiosensitivity, ↑ apoptosis | (120) |
| Chordoma | miR-124-3p, iASPP | MUG-Chor1, U-CH1 | ∆ XIST: ↓ cell proliferation, ↑ apoptosis | (121) |
