# Supplementary material for: X-Inactive-Specific Transcript: Review of Its Functions in the Carcinogenesis
Source: Front Cell Dev Biol. 2021 Jun 11;9:690522. doi: 10.3389/fcell.2021.690522 (PMC8226258; doi:10.3389/fcell.2021.690522)
Supplement: Supplementary file 2 [file Table_2.docx]

**Supplementary Table S2** - Summary of studies that assessed expression of XIST in clinical samples (ANTs: adjacent normal tissues, RFS: Recurrence-free survival, FFPE: Formalin-fixed, paraffin-embedded, OS: Overall survival, 5FU: 5-fluorouracil, Dox: Doxorubicin, DFS Disease-free survival, PFS: progression-free survival, GEO: Gene Expression Omnibus, PTBE: Peritumoral brain edema, NBTs: normal brain tissues, TNBC: Triple-negative breast cancer, LAD: lung adenocarcinoma, EVs: Extracellular vesicles).

| Cancer type | Numbers of clinical samples | Expression  (Tumor vs. Normal) | Kaplan-Meier analysis | Univariate cox regression | Multivariate cox regression | Reference |
| --- | --- | --- | --- | --- | --- | --- |
| Bladder cancer | 67 pairs of BC tissues and matched ANTs | Up | - | - | - | (32) |
|  | 52 BC tissues and matched ANTs | Up | High expression level of XIST was significantly correlated with shorter OS. | - | - | (33) |
|  | 52 BC tissues and ANTs | Up | High expression level of XIST was significantly associated with shorter OS. | - | - | (35) |
| Esophageal squamous cell carcinoma | 78 ESCC tissues and non-tumor tissues | Up | - | - | - | (55) |
|  | 127 pairs of ESCC tissues and ANTs | Up | High XIST expression predicted poor prognosis of ESCC patients. | XIST expression level was significantly associated with OS of ESCC patients. | XIST expression level was independent prognostic factor for ESCC patients. | (56) |
|  | 89 ESCC tissues and ANTs | Up | Patients with high XIST expression had a shorter OS. | OS was linked with XIST expression. | High XIST expression was an unfavorable prognostic factor along with TNM stage. | (122) |
| Nasopharyngeal carcinoma | 108 pairs of NPC tissues and ANTs | Up | XIST high was significantly associated with a worse OS. | - | XIST was an independent risk factor for prognosis. | (47) |
|  | 20 NPC tissue samples and 10 normal nasopharyngeal epithelial specimens | Up | - | - | - | (48) |
|  | 25 pairs of NPC tissues and ANTs | Up | - | - | - | (49) |
|  | 40 NPC tissues and matched ANTs | Up | - | - | - | (51) |
| Laryngeal squamous cell carcinoma | 40 LSCC tissues and matched ANTs | Up | High level of XIST was associated with the low survival rate of LSCC patients. | - | - | (52) |
|  | 34 LSCC and ANTs | Up | - | - | - | (53) |
| Oral squamous cell carcinoma | 15 pairs of OSCC tumor tissues and ANTs | Down | - | - | - | (54) |
| Gastric cancer | 65 GC tissues and the ANTs  45 patients with metastasis and 20 patients without metastasis | Up | - | - | - | (12) |
|  | 98 GC and ANTs | Up | Higher expression of XIST was associated with a shorter OS in GC patients. | - | - | (13) |
|  | 106 fresh-frozen GC and ANTs | Up | Patients with higher expression of XIST tended to have worse OS. | XIST expression level was significantly associated with OS of GC patients. | XIST expression level was independent prognostic factor for patients with GC. | (16) |
|  | 98 GC tissues and ANTs | Up | High expression of XIST was related to the poor prognosis of GC. | - | - | (57) |
| Early gastric cancer | 76 pairs of EGC tissues and ANTs and EGC plasma | Up | - | - | - | (15) |
| Colorectal cancer | 120 cancer tissues and ANTs | Up | Patients with higher expression of XIST had poor OS. | - | - | (17) |
|  | 70 primary CRC tissues from patients showing objective response to 5FU treatment and 70 from patients showing no response.  120 serum specimens from CRC responding and non-responding patients to 5FU treatment | Up | Patients with higher expression of XIST indicated a relatively poor OS rate and RFS rate. | XIST level was identified as potential independent prognostic factors for OS of CRC patients receiving 5FU treatment. | XIST overexpression was an independent prognostic factor in CRC patients receiving 5FU treatment.  . | (18) |
|  | 44 pairs of CRC tissues and ANTs | Up | - | - | - | (19) |
|  | 36 CRC tissues and ANTs | Up | - | - | - | (58) |
|  | 31 advanced CRC patients: 13 DOX-sensitive CRC tissues and 18 DOX-resistant CRC tissues | Up | - | - | - | (59) |
|  | 317 pairs of CRC tissues and ANTs | Up | Higher XIST expression was correlated with poor OS rate of CRC patients. | Higher XIST expression was an independent predictor for larger tumor size, higher TNM stage, serous membrane infiltration, and lymphatic metastasis of CRC patients. | Higher XIST expression was an undesirable prognostic factor in CRC patients. | (21) |
|  | 20 CRC patients and matched ANTs | Up | - | - | - | (22) |
|  | 294 pairs of CRC tissues and ANTs | Up | Patients with higher XIST expression displayed poorer prognosis. | Higher XIST expression was an undesirable prognostic factor in CRC patients. | Higher XIST expression was an undesirable prognostic factor in CRC patients. | (60) |
|  | 60 CRC tissues and matched ANTs | Up | High XIST expression was correlated with a shorter DFS in CRC patients. | - | - | (23) |
|  | 196 CRC tissues and corresponding ANTs | Up | High expression of XIST predicts poor PFS and poor OS in CRC patients. | High XIST expression was risk factor of poor OS in CRC patients. | High XIST expression was independent risk factor for poor OS in CRC patients. | (123) |
|  | serum EVs from 41 healthy participants and 94 CRC patients | Up | Patients with higher expres­sion of lncRNA XIST in serum EVs had a worse 5-year survival rate and a short­er life cycle. | - | - | (124) |
|  | 115 CRC tissues and matched ANTs | Up | Patients with higher lncRNA XIST expression had worse OS. | - | XIST expression was independent prognostic factor for CRC patients. | (20) |
| Pancreatic cancer | 139 pairs of PC and corresponding ANTs | Up | High expression level of XIST was correlated with poor prognosis. | - | - | (24) |
|  | 40 pairs of PC tissues and ANTs | Up | - | - | - | (25) |
|  | 30 pairs of tumor tissues and ANTs | Up | - | - | - | (26) |
|  | 120 PC tissues and matched ANTs | Up | - | - | - | (28) |
|  | 73 pairs of primary PC tissues and matched ANTs | Up | Patients with higher XIST expression had a significantly shorter OS and DFS. | - | - | (27) |
|  | 25 pairs of PC tissues and ANTs | Up | - | - | - | (30) |
|  | 64 pairs of PC tissues and the corresponding ANTs | Up | Higher expression of XIST was associated with decreased OS in PC patients. | XIST expression and was an independent predictors of PC prognosis. | XIST overexpression was an independent poor prognosis risk factor. | (29) |
| Hepatocellular carcinoma | 52 pairs of HCC tumor tissues and corresponding ANTs | Up | Patients with high XIST expression had a poorer survival rate. | - | - | (61) |
|  | 55 HCC tissues and ANTs | Down | - | - | - | (62) |
|  | 88 HCC and ANTs | Up | Higher expression of XIST was associated with a shorter DFS in patients with HCC. | - | - | (63) |
|  | 77 HCC tissues and matched ANTs | Down | - | - | - | (64) |
|  | 40 pairs of HCC specimens and ANTs | Down | - | - | - | (65) |
|  | 68 HCC tumor and ANTs | Down | Patients with low XIST expression tended to have shorter OS. XIST expression resulted in significantly poor OS of HCC. | - | XIST expression level was independent prognostic factor for HCC OS rates. | (125) |
|  | 28 pairs of female HCC tissues and ANTs | Down | - | - | - | (66) |
| Renal cell carcinoma | 50 RCC tissue samples and matched ANTs | Down | - | - | - | (67) |
|  | 26 pairs of RCC tissue samples and ANTs | Up | - | - | - | (68) |
| Lung cancer | 28 pairs of primary lung cancer and matched ANTs | Up | - | - | - | (41) |
|  | 42 LAD and matched-normal tissue samples from patients who had been treated with Cisplatin | Up | - | - | - | (42) |
|  | 33 NSCLC tissue and corresponding adjacent non-tumor tissue samples | Up | - | - | - | (69) |
|  | 17 NSCLC tissue samples and ANTs | Up | - | - | - | (44) |
|  | 33 NSCLC tissue specimens and ANTs | Up | Higher expression of XIST is associated with shorter OS rate. | - | - | (71) |
|  | 30 NSCLC specimens and matched ANTs | Up | - | - | - | (72) |
|  | 15 NSCLC and ANTs | Up | - | - | - | (74) |
|  | 96 pairs of NSCLC tumor tissues and ANTs | Up | High XIST expression was associated with shorter OS and DFS. | XIST was an independent risk factor for poor prognosis in NSCLC patients. | XIST overexpression was an independent poor prognosis risk factor for NSCLC patients. | (75) |
|  | 53 pairs of fresh NSCLC and ANTs | Up | Higher XIST mRNA expression is correlated with an improvement of the PFS in NSCLC patients. | - | XIST overexpression was an independent predictor of OS in NSCLC patients. | (43) |
|  | 24 NSCLC tissues from DDP-sensitive patients and 30 tissues from DDP-resistant patients and 25 normal lung tissue controls | Up | - | - | - | (76) |
|  | 30 NSCLC tissues and ANTs | Up | - | - | - | (77) |
|  | 40 lymph node metastasis tissues and non-metastasis tissues | Up | - | - | - | (45) |
|  | 30 pairs of NSCLC and ANTs | Up | - | - | - | (78) |
|  | 45 pairs of NSCLC and ANTs | Up | High XIST expression was associated with shorter OS. | - | - | (79) |
|  | 32 pairs of NSCLC tumor tissues and corresponding non-tumor tissues  64 serum samples: 32 NSCLC patients and 32 normal controls | Up | -- | -- | -- | (126) |
|  | 156 pairs of NSCLC and corresponding ANTs | Up | Patients with increased XIST expression experienced poor OS. | - | XIST expression level was an independent factor in predicting the OS of NSCLC patients. | (127) |
|  | 50 pairs of NSCLC tissues and ANTs | Up | - | - | - | (80) |
| Breast cancer | 54 breast cancer tissues and matched ANTs | Up | - | - | - | (9) |
|  | 35 primary TNBC tissues and ANTs | Down | - | - | - | (8) |
|  | 710 patients with breast cancer from GEO database  11 primary tumors and 13 brain metastatic tumors | Down | Low level of XIST was associated with a poor brain metastasis-free survival, but not bones metastasis–free survival. | - | - | (81) |
|  | 40 BC tissues and ANTs | Down | - | - | - | (82) |
|  | 63 metastatic, 79 tumor and 20 normal breast tissues | Down | - | - | - | (83) |
|  | Tissue samples: 90 BC and ANTs  Blood samples: 36 BC patients and 32 healthy controls | Down | High expression level of XIST could lead to good OS and RFS for breast cancer patients. | - | XIST indicated favorable prognostic impact for breast cancer patients. | (84) |
|  | 30 pairs of BC tissues and adjacent non-tumor tissues | Down | - | - | - | (7) |
| Ovarian cancer | 98 pairs of EOC tissues and ANTs | Up | Patients with high XIST expression had shorter PFS or OS. | Prognosis of EOC patients was closely related to the expression level of XIST. | High XIST expression is an independent predictor of prognosis in patients with EOC. | (87) |
|  | 87 ovary cancer tissues and ANTs | Down | Patients with high XIST expression had significantly higher 5-year tumor-free survival rate. | - | - | (88) |
|  | 25 pairs of tumor tissues and ANTs | Down | - | - | - | (89) |
| Prostate cancer | 62 PCa specimens and ANTs | Down | Patients with low expression of XIST showed poor OS. | - | - | (90) |
| Osteosarcoma | 30 pairs of OS specimen and ANTs | Up | - | - | - | (99) |
|  | 35 OS tissues and matched ANTs | Up | - | - | - | (92) |
|  | Fresh frozen OS samples and corresponding ANTs | Up | - | - | - | (94) |
|  | 50 pairs of OS tumor tissues and ANTs | Down | Patients with high XIST expression had significantly longer OS. | - | - | (93) |
|  | 40 pairs of OS tissues and matched ANTs | Up | High expression of XIST was inversely correlated with the OS rate. | - | - | (96) |
|  | 20 pairs of tissues and ANTs | Up | - | - | - | (97) |
|  | 64 pairs of OS tissues and ANTs | Up | Patients in the high XIST expression group had a worse OS. | - | - | (98) |
|  | 66 pairs of fresh-frozen OS tissues and ANTs | Up | - | - | - | (100) |
|  | 145 OS tissues and ANTs | Up | High expression of XIST was associated with poor OS. | XIST expression was an independent prognostic factor for the survival. | XIST expression was an independent prognostic marker for the survival of OS patients. | (91) |
| Glioma | 34 tumor samples and 13 normal samples,  12 tumor samples and 7 normal samples obtained from GEO database | Up | - | - | - | (36) |
|  | 55 primary glioma tissues and 10 normal brain tissues | Up | - | - | - | (38) |
|  | 69 pairs of glioma tissues and PTBE tissues | Up | High XIST expression was related shorter OS time. | XIST expression caused significant differences in survival time. | High XIST expression was of high risk. | (40) |
|  | 180 samples from GEO database: 23 non-tumor, 26 astrocytoma, 50 oligodendroglioma, 81 glioblastoma samples | Up | - | - | - | (39) |
|  | 30 glioma tissues and 18 normal brain tissues | Up | - | - | - | (101) |
|  | 8 glioma tissues and NBTs | Up | - | - | - | (102) |
|  | 30 glioma patients and 30 normal controls | Up | - | - | - | (103) |
| Thyroid cancer | 77 pairs of thyroid cancer and non-tumor tissue samples | Up | Patients with high expression of XIST had a shorter survival time. Higher XIST expression was association with poorer prognosis in patients with thyroid cancer. | XIST expression caused differences in OS time. | Differences in OS time caused by XIST expression were significant. | (105) |
| Acute myeloid leukemia | 62 bone marrow samples from AML patients and 20 healthy controls | Up | - | - | - | (108) |
| Papillary thyroid carcinoma | 24 pairs of PTC and ANTs | Up | - | - | - | (106) |
|  | 36 pairs of PTC tissues and ANTs | Up | - | - | - | (107) |
| Retinoblastoma | 30 RB specimens and normal retinal tissues | Up | - | - | - | (109) |
|  | 20 RB tissues and 8 normal retinas | Up | - | - | - | (110) |
|  | 35 RB samples and 7 normal retina samples | Up | - | - | - | (111) |
|  | 25 RB tissues and 6 matched normal retinal tissues | Up | - | - | - | (112) |
|  | 42 RB specimens 40 healthy control | Up | - | - | - | (113) |
| Cervical cancer | 30 pairs of non-tumor adjacent tissue samples | Up | - | - | - | (114) |
|  | 35 CC tissues and normal tissues | Up | Patients with high XIST expression had significantly shorter OS. | - | - | (115) |
|  | 52 pairs of CC tissues and matched normal tissues | Up | Patients with high expression of XIST had a lower survival rate. Upregulated XIST was an unfavorable prognostic factor for CC patients. | - | - | (116) |
|  | 49 FFPE tissue samples from patients treated with chemoradiation therapy. | Up | XIST expression levels were significantly associated with OS rates. | - | XIST expression levels may be a potential prognostic factor for cervical cancer OS rates. | (128) |
| Pituitary neuroendocrine tumor | 51 invasive PitNET tissues, 23 normal pituitary tissues and 35 non-invasive PitNET tissues | Up | - | - | - | (117) |
| Malignant melanoma | 60 pairs of primary MM tissues and ANTs  Oxaliplatin resistant samples and oxaliplatin sensitive samples | Up | Patients with high expression of XIST had poor OS. | - | - | (118) |
| Neuroblastoma | 30 NB tissues and 30 normal adrenal tissues | Up | - | - | - | (119) |
|  | 36 NB tissues and 20 normal adrenal tissues | Up | - | - | - | (120) |
| Chordoma | 38 tumor tissues and 15 normal tissues | Up | High lncRNA XIST expression was associated with decreased OS rate. | - | - | (121) |
